# Decreased Pituitary Height and Stunted Linear Growth After Radiotherapy in Survivors of Childhood Nasopharyngeal Carcinoma Cases

**DOI:** 10.3389/fendo.2018.00643

**Published:** 2018-11-19

**Authors:** Chuanbo Xie, Jiaoxing Li, Zijin Weng, Long-Jun He, Shaohan Yin, Jingwen Zhang, Jing Zhang, Tao Sun, Haojiang Li, Yuying Liu

**Affiliations:** ^1^State Key Laboratory of Oncology in South China, Guangdong Key Laboratory of Nasopharyngeal Carcinoma Diagnosis and Therapy, Department of Cancer Prevention Research, Collaborative Innovation Center for Cancer Medicine, Sun Yat-sen University Cancer Center, Guangzhou, China; ^2^Department of Neurology, First Affiliated Hospital, Sun Yat-Sen University, Guangzhou, China; ^3^Department of Pathology, The Third Affiliated Hospital of Sun Yat-sen University, Guangzhou, China; ^4^State Key Laboratory of Oncology in South China, Guangdong Key Laboratory of Nasopharyngeal Carcinoma Diagnosis and Therapy, Department of Endoscopy, Collaborative Innovation Center for Cancer Medicine, y, Guangzhou, China; ^5^State Key Laboratory of Oncology in South China, Department of Medical Imaging, Collaborative Innovation Center for Cancer Medicine, Guangdong Key Laboratory of Nasopharyngeal Carcinoma Diagnosis and Therapy, Sun Yat-sen University Cancer Center, Guangzhou, China; ^6^School of Public Health, Sun Yat-sen University, Guangzhou, China; ^7^Department of Maternal and Child Health, School of Public Health, Sun Yat-sen University, Guangzhou, China; ^8^State Key Laboratory of Oncology in South China, Guangdong Key Laboratory of Nasopharyngeal Carcinoma Diagnosis and Therapy, Department of Cancer Prevention, Collaborative Innovation Center for Cancer Medicine, Sun Yat-sen University Cancer Center, Guangzhou, China

**Keywords:** pituitary gland, childhood nasopharyngeal carcinoma, radiotherapy, growth hormone deficiency, linear growth

## Abstract

To examine the morphological changes of the pituitary glands and linear growth of childhood nasopharyngeal carcinoma (NPC) cases who accepted radiotherapy. A total of 90 children (i.e., age less than 18 years) who were diagnosed as NPC at Sun Yat-sen University Cancer Center from January 2009 to January 2016 were identified by reviewing medical records. Two radiologists reviewed and measured the pre-radiation, post-radiation, and the latest available pituitary gland heights independently. Patients' current height information was collected by telephone interviews. We compared the pituitary height differences using paired *t*-tests and estimated the pituitary height trajectories within each sex by mixed regression models. Height-for-age Z-score was calculated for each patient using the WHO growth reference data for 5–19 years as reference. Most of the included participants were of male sex (75.6%) and over half were diagnosed at stage IV (58.4%). Among the 90 included participants, 89 had one repeated measurement of the pituitary height and 79 had two repeated measurements of the pituitary height. Seventy six of the 89 childhood NPC participants had reduced pituitary heights after radiation and accounted for 85.4% of the whole population. The means of the pituitary heights before and after radiotherapy were 6.4 ± 1.3 mm and 5.6 ± 1.2 mm (*P* < 0.001), respectively. The mean of height-for-age Z-score for childhood NPC cases was significantly below zero (−0.54, 95% CI = −0.74, −0.34). We concluded that childhood NPC cases had decreased pituitary heights and stunted linear growth after radiotherapy.

## Introduction

Nasopharyngeal carcinoma (NPC) is a rare malignant disease in childhood. It accounts for 0.1 to 2.3 percent of all NPC cases in China (1). Compared to their adult counterparts, childhood NPC cases were more likely to be diagnosed at advanced stages but with superior treatment outcomes (2). The 5-year overall survival rate for childhood NPC cases was over 80% and the cure rate ranged from 30 to 60% (3). Currently, radiotherapy and chemotherapy are the commonly used methods for treatment of childhood NPC (4). However, when radiation beam kills the nasopharyngeal carcinoma cells, it might also damage the nearby cranial tissues and cause severe side effects.

Pituitary gland located at the roof of the nasopharynx secretes growth hormones and plays important role in maintaining normal childhood physical growth (5). Due to the anatomical proximity, the pituitary gland is very likely to be affected by the radiotherapy for the nasopharynx. Several studies have confirmed that children with acute lymphoblastic leukemia (ALL) who received radiotherapy had significant higher risk of developing growth hormone deficiency and lower final height than their normal counterparts even after growth hormone treatment (6, 7). However, few studies aimed at determining the morphology changes of the pituitary gland and linear growth after radiotherapy in children with solid tumors, which require much higher radiation dosage than non-solid tumors such as ALL (8, 9).

In this study, we aim to examine the morphological changes of the pituitary gland of childhood NPC cases before and after radiotherapy using the pituitary height as a proxy of its volume and to investigate the long-term impact of radiotherapy on childhood NPC cases' linear growth in a selected sample of childhood NPC cases in China. Our study had implications in reminding the radiologist to balance the pros and cons of high dosage of radiation related treatment outcomes and optimize the radiotherapy for childhood NPC cases. It could also shed light on explaining the mechanism of radiation related growth retardation.

## Materials and methods

A total of 109 children (i.e., age less than 18 years) who were diagnosed as NPC at Department of Nasopharyngeal Carcinoma at Sun Yat-sen University Cancer Center from January 2009 to January 2016 were identified by reviewing medical records. Among the 109 cases, 19 were excluded out of analysis because of missing clinical information, without MRI images, or unavailable pituitary heights at enrollment. At last 90 cases were included into analysis.

Two radiologists (i.e., Haojiang Li and Shaohan Yin) reviewed and directly measured the patients' pre-radiation, post-radiation, and the latest available pituitary gland heights from the midline sagittal scan series on MRI images. We used the average of the two radiologists' measured heights as the final pituitary gland height. Patients' current height information (by August 2018) was collected via phone interview.

We used means and standard deviations to describe the continuous variables and percentages to describe categorical variables. Paired *t*-tests were used to examine the difference between pre-radiation and post-radiation pituitary heights. In order to explore the changes of the pituitary heights with time, we used mixed regression models to estimate the pituitary heights at time points before, 1 months, and 3 months after radiotherapy within each stratum of sex. Then, we compared the estimated pituitary heights at pre-radiation and 3 months after radiation within each stratum of sex using estimating procedure. Finally, the interaction between time after radiotherapy and sex on pituitary height was tested by adding the product term of “time × sex” into the mixed regression model. WHO growth reference data for 5–19 years was used to calculate the height-for-age Z-score and independent one sample *t*-test was used to test whether the height-for-age Z-score was below zero. All the tests were two sided and 0.05 was set as the significant level. We performed the data analyse with SAS 9.3 software (SAS Institute, Cary, NC).

This study had been approved by the Institute Review Board of Sun Yat-sen University Cancer Center (GZR2017-100). Since this study was based on medical records review and no personal identifiers were used, informed consent forms were not required.

## Results

### Patients' characteristics

The mean age of the 90 included childhood NPC cases was 15.2 years (*SD* = 2.6 years). Most of them were of male sex (75.6%) and over half were diagnosed at stage IV (58.4%). Among the 90 included participants, 89 had one repeated measurement of the pituitary heights and 79 had two repeated measurements of the pituitary heights. The median follow-up time for the measurement of pituitary heights was 167 days (quartile range 104–195 days). Seventy patients (77.8%) self-reported their height information through phone interview. The mean age of the patients at reporting their heights was 19.7 years (*SD* = 3.6 years).

### The morphological changes of pituitary on magnetic resonance imaging (MRI) images

**Figure 1:**
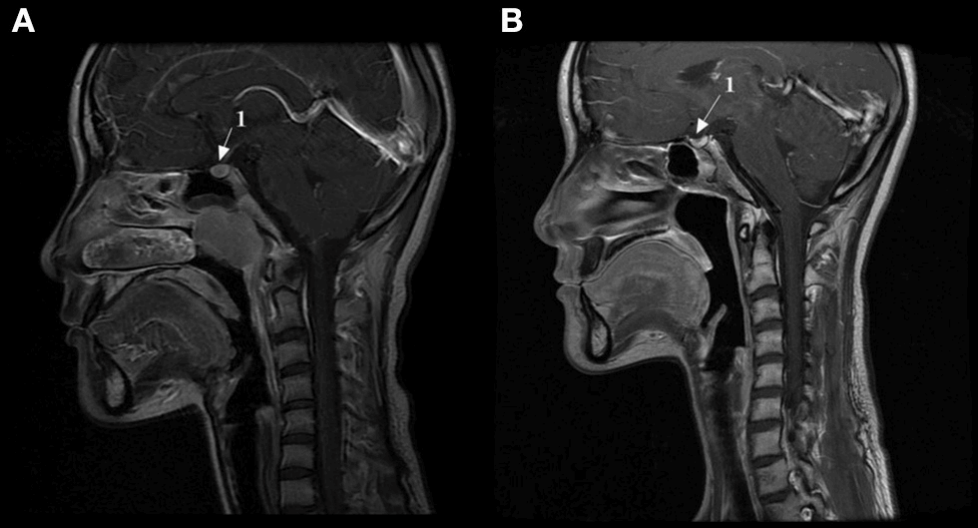
The MRI images of the pituitary gland of one of the childhood NPC patients before and after radiotherapy. **(A)** MRI image of the pituitary gland before radiotherapy. **(B)** MRI image of the pituitary gland after radiotherapy. 1, Pituitary gland.

The typical change of the pituitary gland after radiotherapy was height reduction. Seventy six of the 89 childhood NPC participants had reduced pituitary height after radiation and accounted for 85.4% of the whole population. We observed that some of the patients even had empty sella syndrome after radiotherapy. Figure 1 showed the MRI images of the pituitary gland of one of the childhood NPC cases before (A) and after (B) radiotherapy.

### Pituitary changes and linear growth after radiotherapy

**Figure 2:**
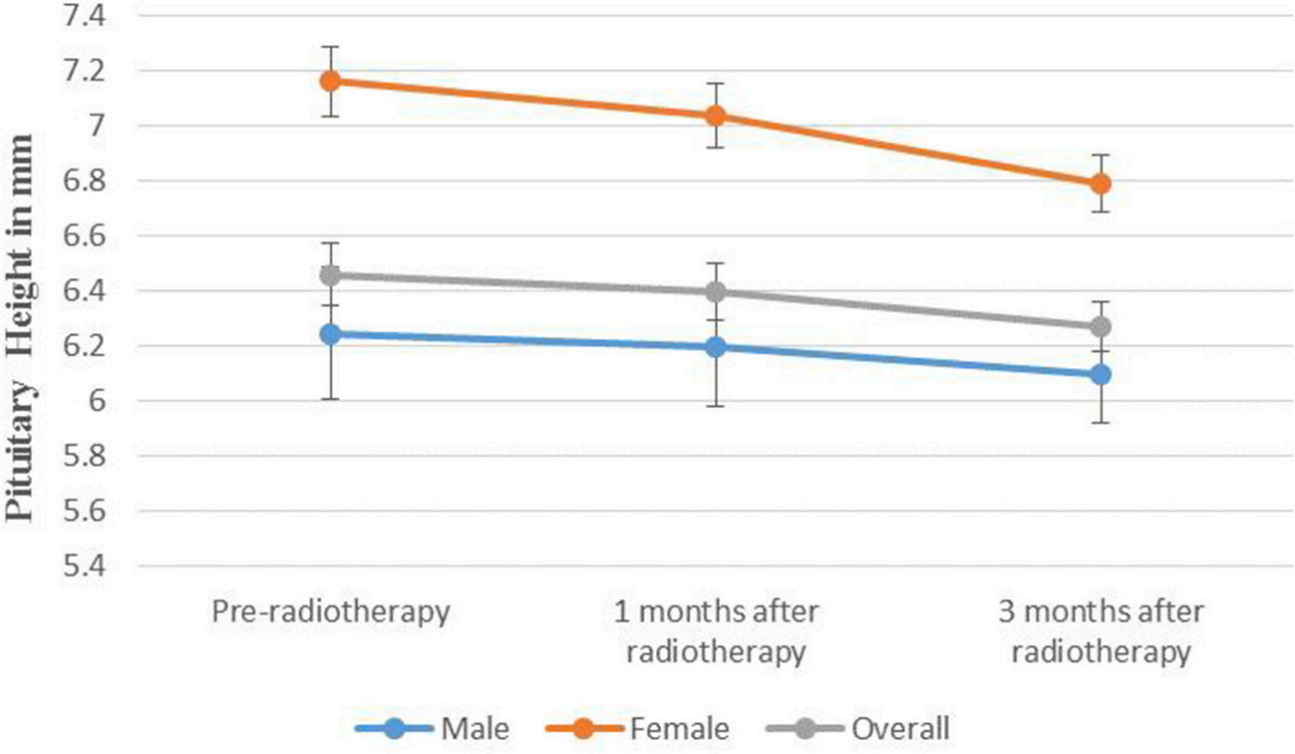
The estimated pituitary heights of childhood nasopharyngeal carcinoma cases before and after radiotherapy by gender.

The mean pituitary heights before and after radiotherapy were 6.4 mm (*SD* = 1.3 mm) and 5.6 mm (*SD* = 1.2 mm), respectively (paired *t*-test, *P* < 0.001). Female patients had significantly higher pre-treatment pituitary heights than their male counterparts (6.8 mm vs. 6.2 mm, *P* = 0.044). The overall estimated pituitary heights for the 90 included participants at time points before radiotherapy, 1 months, and 3 months after radiotherapy were 6.5, 6.4, and 6.3 mm, respectively (Figure 2). The estimated pituitary heights for female patients and male patients at time points before radiotherapy, 1 months, and 3 months after radiotherapy were 7.2, 7.0, and 6.8 mm and 6.3, 6.2, and 6.1 mm, respectively (Figure 2). The difference in pituitary height before and 3 months after radiotherapy were 0.37 mm (*P* = 0.001) and 0.15 mm (*P* = 0.001) for female and male patients, respectively. Our interaction analysis suggested that there were marginally significant interaction between days in cohort and gender on pituitary heights (*P* = 0.072). The mean of the height-for-age Z-score for childhood NPC cases was significantly deviated from zero (−0.54, 95% CI = −0.74, −0.34, *P* < 0.001). There was no significant difference in height-for-age Z-score between male and female patients (−0.58, 95% CI = −0.81, −0.36 vs. −0.43, 95% CI = −0.89, 0.05, *P* = 0.485).

## Discussion

To the best of our knowledge, this is the first study to examine the morphological changes of the pituitaries and linear growth among childhood NPC cases who accepted radiotherapy. We found that the pituitary height, which is a reliable representative of the pituitary volume (10), was significantly reduced after radiotherapy among childhood NPC cases and this change have long term side effects on their linear growth.

Pituitary is the most important organ for secreting growth hormones and sustaining children's thrive and childhood is the crucial period for pituitary gland's growth and development. Once the pituitary is damaged by external environmental factors such as radiation at this venerable period, it will be very hard to recover. Similar with the findings from ALL patients (11), in this study we found that the pituitary heights significantly reduced among childhood NPC cases which could be explained by following mechanisms. First, the pituitary cells undergo apoptosis after cranial radiation and thus the total number of the pituitary cells reduced (12). Second, cranial radiation might affect the vascular function and the blood and oxygen supply of the pituitary gland and thus lead to cell death and reduced height of the pituitary gland (13). Although the threshold of the dosage of radiation in triggering the above mechanisms was still controversial, one of the study showed that even low dosage of radiation (i.e., 18 Gy) would disturb the pituitary gland growth (14). Therefore, the cranial radiation dosage for treatment of childhood NPC which is much higher than that of non-solid tumors (i.e., 52–70 Gy) definitely will lead to morphological change of the pituitary gland and related hormone disorders.

We observed stunted linear growth for childhood NPC cases after radiotherapy which in line with the findings of a previous study which showed that even after growth hormone therapy a majority of childhood cancer patients who accepted cranial radiation will have stunting (i.e., about half) (15). Therefore, it is very important for clinicians taking the long term sides effects of cranial radiation into consideration when they make the radiation plans for childhood NPC cases. If possible, they should use less aggressive dosage of radiation and consider how to perform more target-oriented radiotherapy to avoid damaging pituitary gland and protect childhood NPC cases' growth and height as much as possible.

Our study had several limitations. First, we didn't directly measure the baseline and post-treatment growth hormone levels of the NPC cases, thus we were unsure whether the reduced pituitary height was the true determinant of reduced growth hormone and stunted linear growth. Second, the radiologists didn't observe the pituitary stalks when they measured the pituitary heights for some of the patients. Therefore, the pituitary heights of these patients might subject to measurement error. But the proportion of these patients was small (less than 5%), it was less likely that the measurement error affected our finding. Third, the number of childhood NPC patients especially the female patients included into analysis was rather small. Therefore, we couldn't role out the possibility that our findings. Third, the number ofle out the possibility that our findings was by chance only. Finally, the median follow-up time for the measurement of pituitary heights was around 3 months. Therefore, we didn't know whether the effect of radiotherapy on the morphological change of the pituitary height could be replicated in longer follow up time.

## Conclusion

Pituitary height was significantly reduced after radiation treatment among childhood NPC cases which affect their linear growth and final heights. Clinicians are expected to consider not only the survival outcomes but also the physical growth when they make the radiation plan for childhood NPC cases, given the high survival and cure rates of these patients.

## Data availability statement

The authenticity of this article has been validated by uploading the key raw data onto the Research Data Deposit public platform (www.researchdata.org.cn), with the approval RDD number as RDDA2018000863.

## Author contributions

CX contributed to hypothesis generation, study design, data analysis, result interpretation, and the manuscript drafting and revision. JL and ZW contributed to manuscript revisions. L-JH, JWZ, JZ, and TS contributed to medical records review, data collection, and data cleaning. HL and SY are the radiologists and they contributed to measurement of the pituitary heights. YL contributed to revision of the manuscript and organized the telephone interview for collecting participants' final heights.

### Conflict of interest statement

The authors declare that the research was conducted in the absence of any commercial or financial relationships that could be construed as a potential conflict of interest.
